# Pseudotumor cerebri in patient on leuprolide acetate for central precocious puberty

**DOI:** 10.1186/s13633-020-00092-4

**Published:** 2020-12-03

**Authors:** Anjumanara Anver Omar, Godfrey Nyaga, Lucy N Wainaina Mungai

**Affiliations:** 1 Department of Paediatrics and Child Health-Nairobi, University of Nairobi, Nairobi, Kenya; 2 Optimal Health KMA Centre-Nairobi, Nairobi, Kenya

**Keywords:** Gonadotropin releasing hormone agonist, Central precocious puberty, Leuprolide acetate, Pseudotumor cerebri

## Abstract

**Background:**

Gonadotropin releasing hormone agonists (GnRHa) are well established as a standard of care for the treatment of central precocious puberty (CPP) worldwide. While numerous delivery systems and routes of administration exist, depot intramuscular injections or sustained-release preparations have been most widely used. Leuprolide acetate is well tolerated among children though some can develop some complications.

**Case presentation:**

We present a case report of a 6.5 year old girl with central precocious puberty who developed signs of pseudotumor cerebri after 2 doses of leuprolide acetate 3.75 mg given monthly. Systemic exam and other tests to look for the cause did not yield anything. However, fundoscopy showed marked papilloedema with blurred disc margins. After six weeks’ treatment with acetazolamide and withdrawal of the GRNHa the papilloedema resolved.

**Conclusions:**

If a patient presents with complaints such as headache, nausea, vomiting, and double vision in pediatric patients treated with GnRH analogue one should highly consider the presence of pseudotumor cerebri and fundus examination be performed.

## Background

Central precocious puberty (CPP) refers to premature activation of the hypothalamic–pituitary–gonadal (HPG) axis, resulting in early development of secondary sexual characteristics [1]. Gonadotropin releasing hormone agonists (GnRHa) are well established as a standard of care for the treatment of CPP worldwide [1]. While numerous delivery systems and routes of administration exist, depot intramuscular injections or sustained-release Leuprolide acetate depot, Triptorelin pamoatede pot which are each available as monthly and 3-monthly depot preparations are frequently used. Histrelin acetate implant which is approved for 12 months treatment, has been found to be effective for upto 2 years [1, 2]. Previously, monthly depot GnRHa were most frequently used. However, additional 3-monthly and 6-monthly formulations, as well as subcutaneous implants, have become available over the past ∼10 year [2]. Leuprolide acetate is well tolerated among children though some can develop sterile abscess at the injection site, menopause-like symptoms, headache, emotional disorders, syncope, osteoporosis, vasodilatation, and peripheral edema as adverse effect to the drug [2–4]. Pseudotumor cerebri (PTC) associated with leuprolide acetate as an adverse effect is extremely rare with only few cases reported in literature [3–5].

We present a case report of a girl with central precocious puberty who developed pseudotumor cerebri with visual loss associated with the use of leuprolide acetate a GnRH analogue.

### Case presentation

**Fig. 1:**
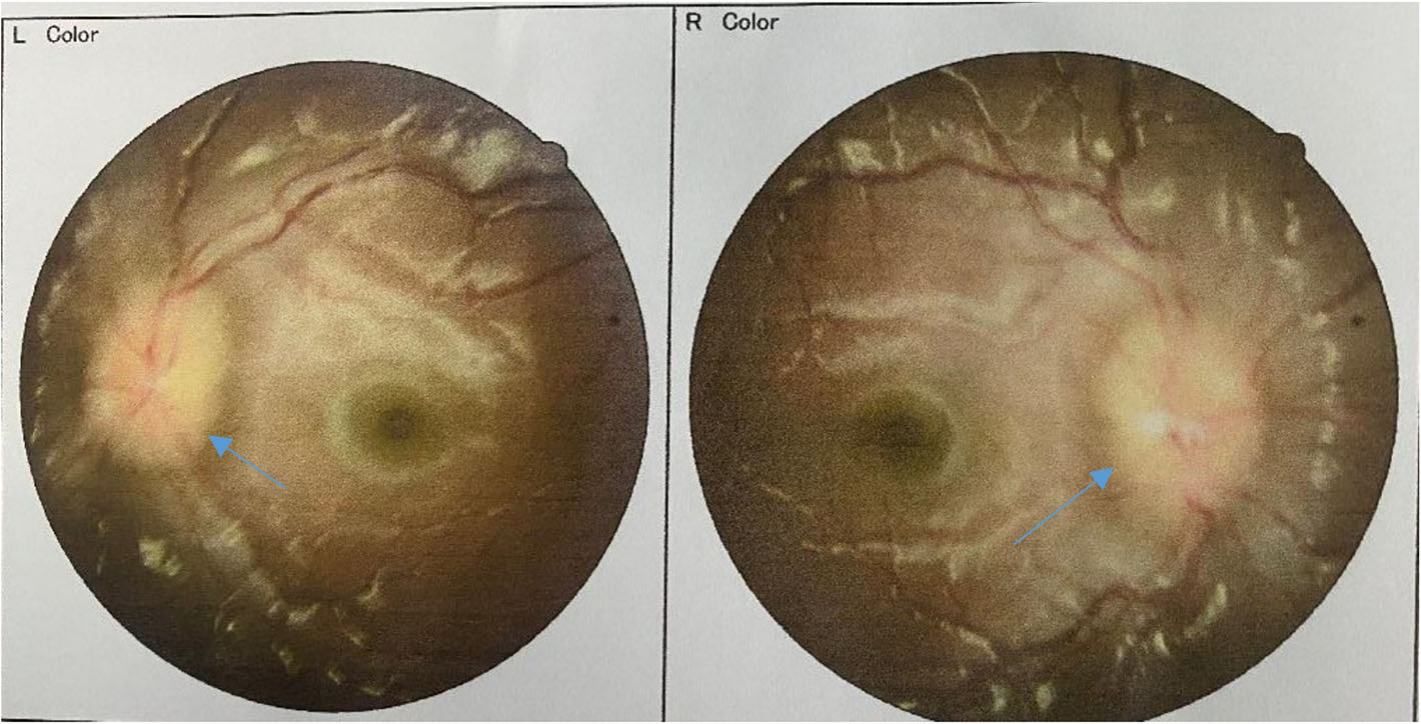
Papilloedema with blurring of the optic disc margins

A 6 year 6 months old female was referred to the Paediatric Endocrinology clinic for breast development and rapid growth for the past 6 months. Prior to the onset of this symptoms she had been well. There was no significant past medical or family history. On examination her vitals were weight 26.5 kg (75th -90th centile), height 125.5 cm (75th -90th centile),blood pressure 90/40 mm/Hg. Pubertal examination revealed left breast at tanner 3 and right breast at tanner 2.Pubic hair was at tanner 1 and no axillary hair noted. Investigations done revealed bone age at 7 years, pelvic ultrasound revealed uterus of 4.2 ml (tanner3),right ovarian volume of 3mls(Tanner 4) and left ovarian volume of 4mls(Tanner 5).Endometrial thickness of 2 mm was also noted. GnRH stimulating test done with leuprolide acetate revealed Luteinizing Hormone (LH) at 7.78mIU/ml and Follicle stimulating Hormone (FSH) at 14.52 mIU/ml. With these findings a diagnosis of precocious puberty was made and patient was started on leuprolide acetate 3.75 mg every 28 days. After 2 doses of leuprolide acetate 3.75 mg patient started developing double vision with partial vision loss. There was no history of headache, vomiting and convulsions. Neurologic and other systemic examination was essentially normal. Patient was referred to ophthalmologist for a funduscopy examination. Ophthalmological examination revealed visual acuity of 6/4.8 in the right eye and 6/4.8 in the left eye. She also had a head tilt to the left with normal extraocular movements. On funduscopy there was severe bilateral Papilloedema with blurring of the optic disc margins. The rest of the fundus was normal (Fig. 1).

**Fig. 2:**
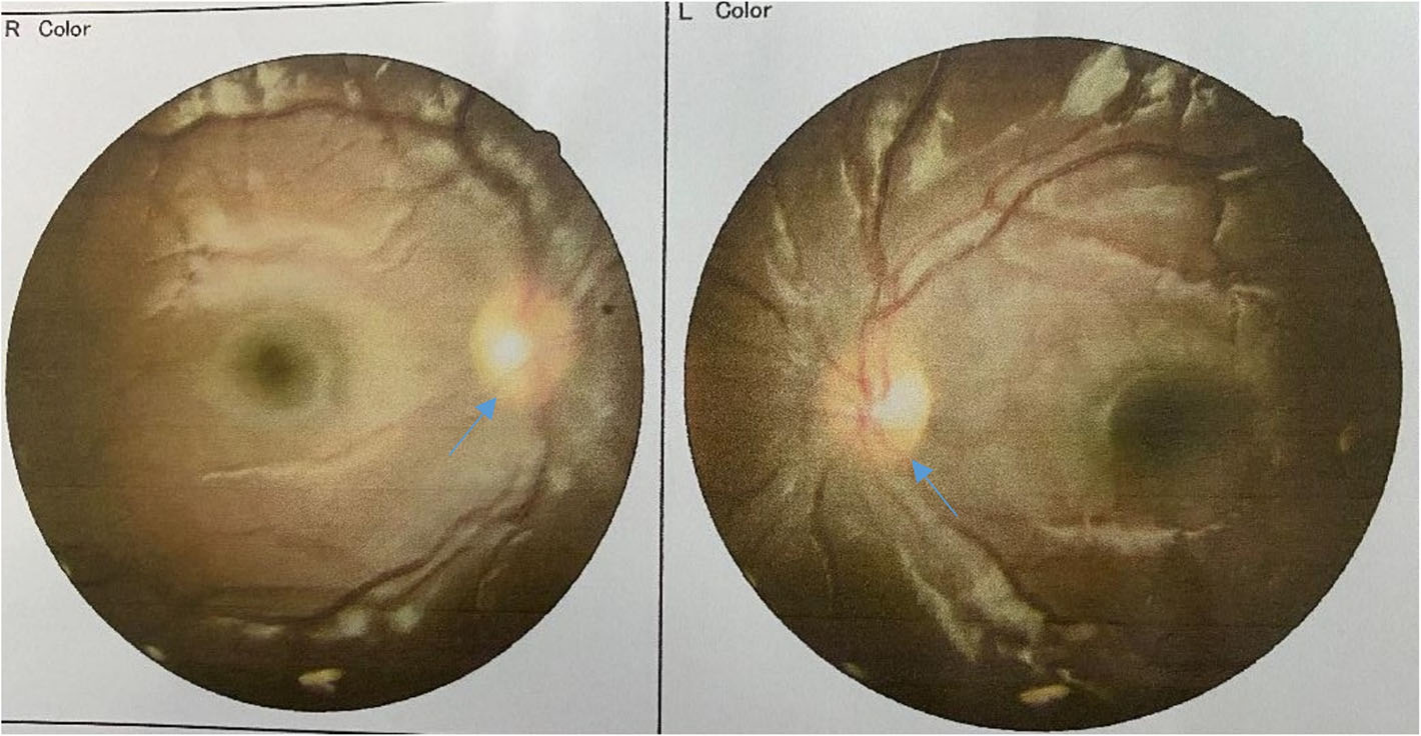
Well defined optic disc margins

Cranial and pituitary Magnetic Resonance Imaging did not reveal any space occupying lesion. Patient was not on any other medications apart from leuprolide acetate. The most likely diagnosis was pseudo tumor cerebri secondary to leuprolide acetate. Because of severe Papilloedema and visual loss leuprolide acetate was discontinued immediately and acetazolamide initiated. After 6 weeks of treatment with acetozalamide the visual acuity as well as papilledema resolved (Fig. 2). Currently patient is off treatment for leuprolide acetate and acetazolamide.

#### Discussion and conclusion

Central precocious puberty (CPP) is amenable to management with GnRH analogues that operate on physiological principles by desensitizing the signaling pathway to gonadotrophin production [6]. The aims of the treatment are to halt and perhaps reverse the secondary sex characteristics of puberty, prevent early onset of menses in girls and attenuate the loss of height potential consequent upon advanced skeletal maturation [1, 2]. Adverse effects of GnRHa therapy are rare, and the associations of most reported adverse events with the GnRHa molecule itself are unclear. Decades of experience have shown that GnRHa treatment is both safe and efficacious. Common side effects mentioned in literature related to use of GnRHa in children are Allergic or local reactions to GnRH injection, withdrawal bleeding, hot flushes, convulsions, slipped capital femoral epiphysis, while prolonged QT interval and Pituitary apoplexy which is rare was reported in adult males, with prostate cancer treated with GnRHa [2, 7]. Pseudotumor cerebri associated to leuprolide acetate a GnRH analogue is an extremely rare event with only few cases reported in the literature.

Pseudotumor cerebri (PTC), is a syndrome that presents with clinical features of elevated intracranial pressure without radiological evidence of an intracranial mass, infection, vascular abnormality, hydrocephalus or changes in the level of consciousness [8–10]. Systemic diseases and drug exposure are associated with pseudotumor cerebri (secondary pseudotumor). PTC in children can present with headache, nausea, vomiting, blurred vision, diplopia, neck stiffness, photophobia, and retro-orbital pain. Our patient presented with partial visual loss and papilledema with no headache after 2 months of leuprolide acetate administration (3.75 mg).Although headache has been reported in 62–91% of cases there are also reports of PTC without headache [11–13]. After stopping the leuprolide acetate the visual acuity as well as papilledema resolved 6 weeks after starting treatment with acetazolamide which is the recommended treatment for PTC [13]. With prompt diagnosis and medical management, most children with mild-to-moderate disc swelling and visual field defects have complete resolution of disc swelling and visual abnormalities [11] as observed in our patient. In our case, after cessation of treatment for precocious puberty, and initiating standard treatment for PTC causing normalization of eye examination highly suggested the cause of GnRH analogues in the pathology. This rare adverse effect of Leuprolide acetate was reported to the distributor company of this drug. The question arises, Should GnRHa therapy be restarted after cessation of symptoms? The answer to this question is unknown. In conclusion if a patient presents with complaints such as headache, nausea, vomiting, and double vision in pediatric patients treated with GnRH analogue one should highly consider the presence of pseudotumor cerebri (PTC) and fundus examination should be performed.

## Data Availability

Data sharing is not applicable to this article as no datasets were generated or analyzed during the current study.

## Abbreviations

GnRH: Gonadotropin Releasing Hormone
GnRHa: Gonadotropin Releasing Hormone analogue
LH: Luteinizing Hormone
FSH: Follicular Stimulating Hormone
CPP: Central Precocious Puberty
PTC: Pseudotumor cerebri

## References

[1] Chen M, Erica A. Eugster. Central Precocious Puberty: Update on Diagnosis and Treatment. Paediatr Drugs. 2015; 17(4): 273–281. doi:10.1007/s40272-015-0130-8. 10.1007/s40272-015-0130-8 PMC5870137 25911294

[2] Krishna KB, Fuqua JS, Rogol AD, Karen O, Klein, JadrankaPopovic CP, Houk, EvangeliaCharmandari PA (2019). Lee. Use of Gonadotropin-Releasing Hormone Analogs in Children: Update by an International Consortium. Horm Res Paediatr.

[3] ÜlküGül A, KaçarBayram M, Kendirci (2016). NihalHatipoğlu, DenizOkdemir, HakanGümüş, SelimKurtoğlu. PseudotumourCerebri Presentation in a Child Under the Gonadotropin-Releasing Hormone Agonist Treatment. J Clin Res Pediatr Endocrinol.

[4] Renato, AntunesSchiaveGermano (2015). Ruth Rocha Franco, SandroMatas and FredericoCasteloMoura. PseudotumorCerebri Associated with Leuprolide Acetate for Central Precocious Puberty-Case Report. J ClinExpOphthalmol.

[5] Boot JH (1996). Pseudotumourcerebri as a side effect of leuprorelin acetate. Ir J Med Sci.

[6] Mul D, Hughes IA (2008). The use of GnRH agonists in precocious puberty. Eur J Endocrinol.

[7] Conteduca V, Di Lorenzo G, Tartarone A, Aieta M (2013). The cardiovascular risk of gonadotropin releasing hormone agonists in men with prostate cancer: an unresolved controversy. Crit Rev Oncol Hematol.

[8] Galgano MA, Deshaies EM. An update on the management of pseudotumorcerebri. ClinNeurolNeurosurg. 2013 Mar;115(3):252–9. 10.1016/j.clineuro.2012.11.018. 10.1016/j.clineuro.2012.11.018 23265564

[9] Per H, Canpolat M, Gümüş H, Poyrazoğlu HG, Yıkılmaz A, Karaküçük S (2013). Clinical spectrum of the pseudotumorcerebri in children: etiological, clinical features, treatment and prognosis. Brain Dev.

[10] Gabriela G. M. Balbi, Sandro L. Matas, Claudio A. Len, Melissa M. Fraga, Iggor O. Sousa, Maria Teresa Terreri. Pseudotumorcerebri in childhood and adolescence: data from a specialized service. Pseudotumor cerebral nainfância e adolescência: dados de um serviçoespecializado . Arq Neuropsiquiatr. 2018;76(11):751–755. 10.1590/0004-282X20180131 30570018

[11] Babikian P, Corbett J, Bell W (1994). Idiopathic intracranial hypertension in children: the Iowa experience. J Child Neurol.

[12] Phillips PH, Repka MX, Lambert SR (1998). Pseudotumorcerebri in children. J AAPOS.

[13] Melissa W, Ko,Grant T, Liu (2010). Pediatric Idiopathic Intracranial Hypertension (PseudotumorCerebri). Horm Res Paediatr.

